# MKK3 Was Involved in Larval Settlement of the Barnacle *Amphibalanus amphitrite* through Activating the Kinase Activity of p38MAPK

**DOI:** 10.1371/journal.pone.0069510

**Published:** 2013-07-29

**Authors:** Gen Zhang, Li-Sheng He, Yue Him Wong, Pei-Yuan Qian

**Affiliations:** KAUST Global Collaborative Research Program, Division of Life Science, The Hong Kong University of Science and Technology, Clear Water Bay, Kowloon, Hong Kong SAR, China; Temple University, United States of America

## Abstract

The p38 mitogen-activated protein kinase (p38MAPK) plays a key role in larval settlement of the barnacle *Amphibalanus amphitrite*. To study the signaling pathway associated with p38MAPK during larval settlement, we sought to identify the upstream kinase of p38MAPK. Three MKKs (MKK3, MKK4 and MKK7) and three MAPKs (p38MAPK, ERK and JNK) in *A. amphitrite* were cloned and recombinantly expressed in *E. coli*. Through kinase assays, we found that MKK3, but not MKK4 or MKK7, phosphorylated p38MAPK. Furthermore, MKK3 activity was specific to p38MAPK, as it did not phosphorylate ERK or JNK. To further investigate the functional relationship between MKK3 and p38MAPK *in vivo*, we studied the localization of phospho-MKK3 (pMKK3) and MKK3 by immunostaining. Consistent with the patterns of p38MAPK and phospho-p38MAPK (pp38MAPK), pMKK3 and MKK3 mainly localized to the antennules of the cyprids. Western blot analysis revealed that pMKK3 levels, like pp38MAPK levels, were elevated at cyprid stage, compared to nauplii and juvenile stages. Moreover, pMKK3 levels increased after treatment with adult barnacle crude extracts, suggesting that MKK3 might mediate the stimulatory effects of adult barnacle extracts on the p38MAPK pathway.

## Introduction

The barnacle *Amphibalanus amphitrite* ( = *Balanus amphitrite*) is a ubiquitous and fouling organism that lives in tropical and sub-tropical waters. It has two major life stages: a pelagic stage and a sessile stage. At the end of pelagic stage, barnacle larvae, called cyprids, search for and attach to suitable substrata. Attached cyprids metamorphose into juveniles to enter the sessile stage. Together, the attachment and metamorphosis processes are known as ‘larval settlement’. Since adult barnacles are a detriment to maritime industries, they are widely used as a model organism for biofouling and antifouling studies [1]–[3].

Significant progress has been made on the molecular mechanisms of larval settlement over the recent years. For instance, a glycoprotein called settlement-inducing protein complex (SIPC) was purified from barnacle adults and found to function as a pheromone that induces larval settlement [4]. Moreover, the NO/cGMP pathway [5], endogenous calcium [6], and cAMP [7] are involved in barnacle larval settlement.

Mitogen-activated protein kinase (MAPK) pathways are well-studied in mammalian cells and known to regulate many biological processes, such as cell proliferation, differentiation and death [8], [9]. Each MAPK pathway includes three levels of kinases: MAPK, MAPK kinase (MAPKK or MKK) and MAPKK kinase (MAPKKK or MKKK). A suitable stimulus leads to the phosphorylation and activation of the MAPKKK, which phosphorylates the Ser-X-X-X-Thr (X represents any amino acid residue) motif of MAPKK. In turn, activated MAPKK stimulates MAPK by dually phosphorylating its Thr-X-Tyr motif [10]. To date, three groups of MAPKs have been defined, including the extracellular signal-regulated kinases (ERK), the c-Jun NH_2_-terminal kinases (JNK) and the p38MAP kinases (p38MAPK) [9], [11]. Among them, p38MAPK is the most studied in the context of signaling transduction.

MKK3 is a universal p38MAPK activator [10]. MKK6 was reported to activate p38MAPK only in mammals [12], [13], carps [14] and frogs [15], but not in *Drosophila* or other invertebrates, to the best of our knowledge. A known JNK activator, MKK4, may also activate p38MAPK in certain cellular contexts [16].

Based on temporal expression profiles and inhibition assays, previous studies have shown that p38MAPK is involved in larval settlement of the polychaete *Hydroides elegans* [17] and the barnacle *A. amphitrite* [18]. Moreover, inhibition of p38MAPK abolishes the stimulatory effects of adult extracts on the larval settlement of *A. amphitrite*, which suggests that p38MAPK might mediate this process [18]. However, these limited observations have not implicated a specific signaling pathway associated with p38MAPK and larval settlement. Furthermore, the effects of SIPC on the p38MAPK pathway are still not known.

Here, we sought to identify the upstream activator of p38MAPK in *A. amphitrite* and investigate its role in larval settlement. Starting from the partial sequences in our transcriptome database of *A. amphitrite* [19], we obtained full-length MKKs (MKK3, MKK4 and MKK7) and MAPKs (JNK, ERK) by Rapid Amplification of cDNA Ends (RACE). We expressed these genes and p38MAPK in *E. coli* and performed *in vitro* kinase assays with the recombinant proteins. We found that p38MAPK is specifically activated by MKK3. Furthermore, the activation and localization patterns of p38MAPK and MKK3 were investigated in *A. amphitrite* cyprid larvae.

## Results

### Characterization of MAPK and MKK Protein Sequences in *A. amphitrite*

The complete open reading frames (ORF) of MKK3, MKK4 and MKK7 consist of 343, 395 and 515 amino acid residues, respectively. Compared to MKK4 and MKK3, MKK7 contains two insertions at the N and C termini (Fig. S1A). The canonical regulatory sequence Ser-X-X-X-Thr was conserved in *A. amphitrite* MKKs and located between subdomain VII and VIII (Fig. S1A). The dual phosphorylation motif Thr-X-Tyr was found on all *A. amphitrite* MAPKs between subdomain VII and VIII [18]. The T103 residue in the ATP docking site was conserved in p38MAPK, but not in JNK or ERK (Fig. S1B).

**Figure 1:**
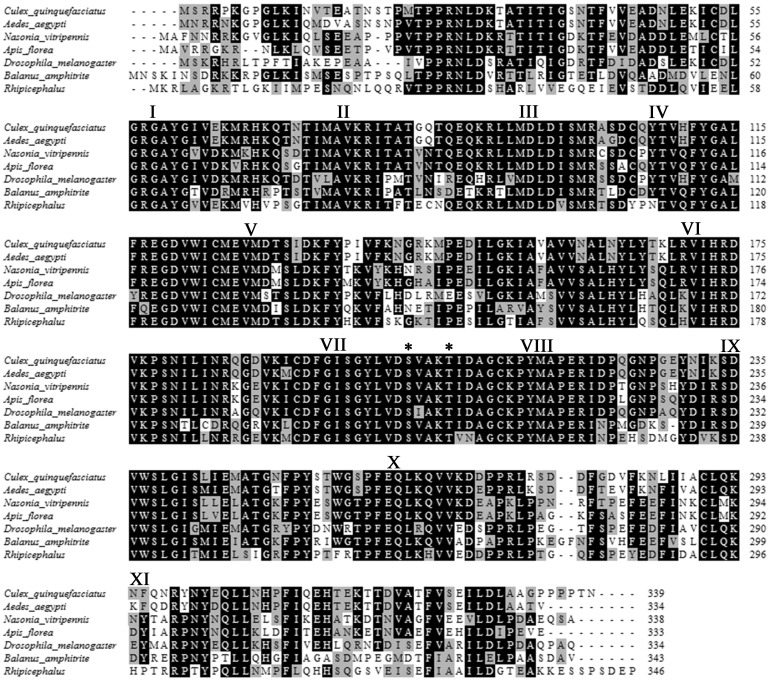
Alignment of MKK3 sequences from different species. *Amphibalanus amphitrite* MKK3 was aligned with those from *Culex quinquefasciatus* (XP001844745.1), *Aedes aegypti* (AAQ68075.1), *Nasonia vitripennis* (XP001604546.1), *Apis florea* (XP003689651.1), *Drosophila melanogaster* (AAF48223.1) and *Rhipicephalus pulchellus* (JAA59480.1). The subdomains I-XI were defined. The conserved dual phosphorylation sites were labeled with asterisks.

**Figure 2:**
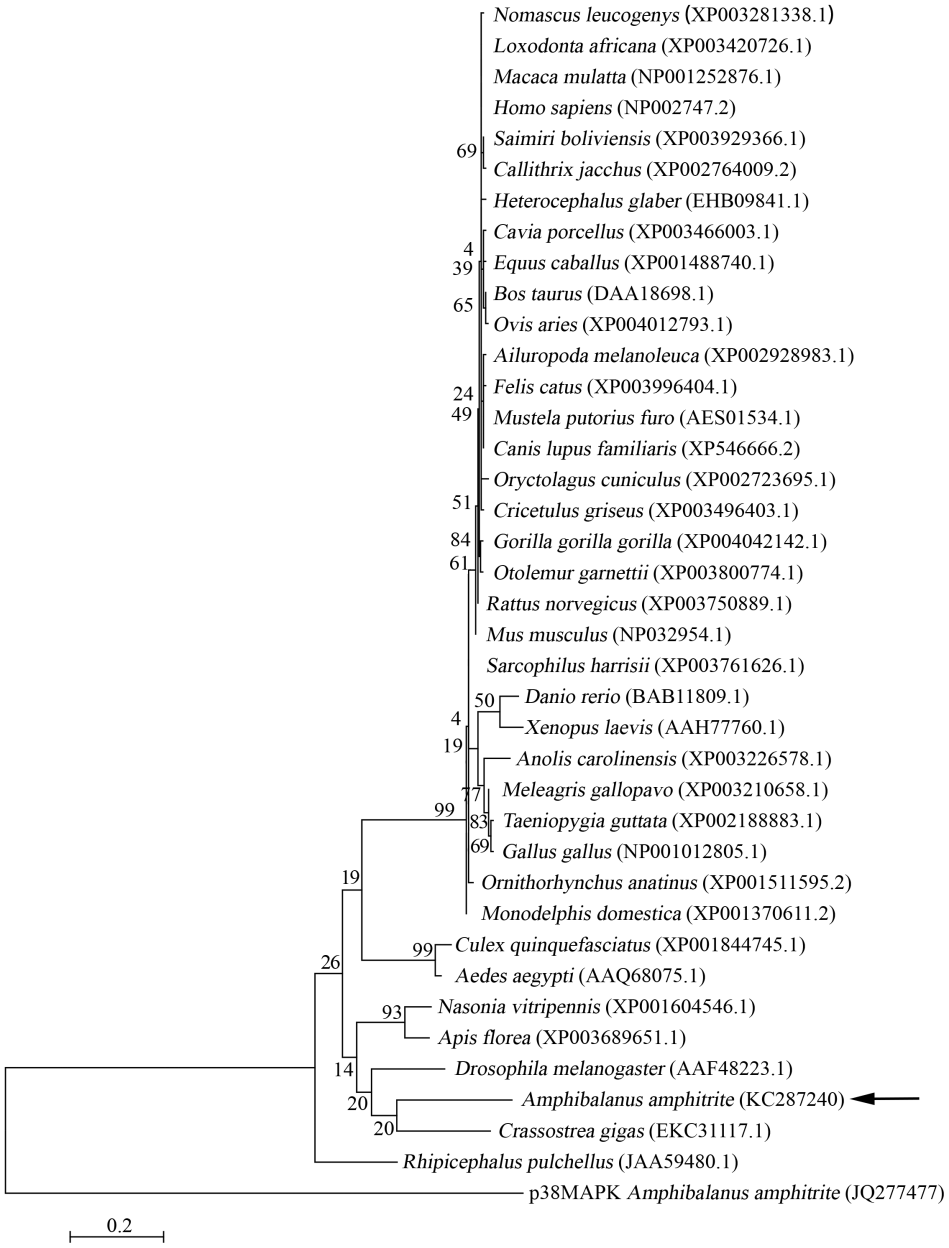
The phylogenetic relationship of MKK3 among different species. Maximum Likelihood (ML) tree was constructed based on the complete amino acid sequences of MKK3 from *Amphibalanus amphitrite* and other species. *A. amphitrite* p38MAPK sequence was chosen as the outgroup. Support for each node was tested with standard bootstrap analysis using 1,000 replications. The Genbank access number for each species was labeled with the species name.

The MKK3 sequences of *A. amphitrite* and other invertebrates were highly similar (Fig. 1). Based on the Maximal Likehood phylogenetic tree, MKK3 from *A. amphitrite* clustered with those of *Crossostrea gigas*, *Drosophila melanogaster*, *Apis florea*, *Nasonia vitripe* and other invertebrates. It was more distantly related to the mammalian MKK3 (Fig. 2). These results suggest that *A. amphitrite* MKK3 is highly similar to MKK3 genes in other invertebrates.

### MKK3 Specifically Phosphorylated p38MAPK

**Figure 3:**
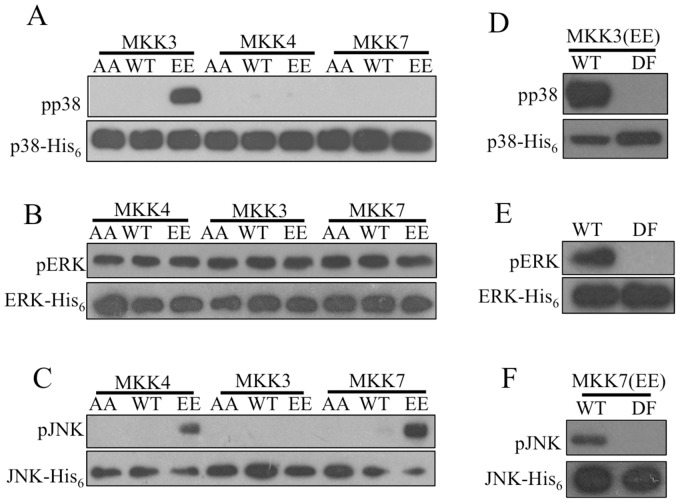
MKK3 specifically phosphorylated p38MAPK. Constitutively activated or inactivated MKKs were constructed by replacing the dual phosphorylation sites with Glu and Ala, respectively. Phosphorylation-defective mutations of MAPKs were constructed by substituting the Thr-X-Tyr motif with Ala-X-Phe. MKKs and MAPKs were expressed in *E. coli* with a GST or His_6_ tag, respectively, and used for kinase assays. (A) Constitutively activated MKK3 effectively phosphorylated p38MAPK. (B) None of the MKKs enhanced activation of ERK, as similar intensity of signal was detected in all lanes. (C) Both constitutively activated MKK4 and MKK7 effectively phosphorylated JNK. (D) MKK3 phosphorylated wild type p38MAPK, but not the phosphorylation-defective mutant. (E) In the absence of MKK protein, wild type ERK was auto-phosphorylated, but no signal was detected when the phosphorylation-defective ERK was used. (F) Constitutively activated MKK7 significantly phosphorylated the wild type form of JNK but not the phosphorylation-defective form. AA: constitutively inactivated mutation; WT: wild type; EE: constitutively activated mutation; DF: phosphorylation-defective mutation.

Constitutively activated and inactivated forms of MKK3, MKK4 and MKK7 were constructed by replacing the dual phosphorylation sites with Glu and Ala, respectively [20]–[23]. Kinase assays revealed that only MKK3 significantly phosphorylated p38MAPK (Fig. 3A). Moreover, MKK3 activity was specific to p38MAPK, as it did not phosphorylate JNK or ERK (Fig. 3B and 3C). Robust phosphorylation of JNK by MKK4 and MKK7 was observed (Fig. 3C). ERK was phosphorylated in all cases, but this likely occurred as a result of auto-phosphorylation [24], [25]. Notably, none of the MKKs increased phosphorylation levels on ERK (Fig. 3B).

To confirm these modifications were occurring on the dual phosphorylation sites, phosphorylation-defective mutants of p38MAPK, JNK and ERK were constructed by replacing their phosphorylation motif Thr-X-Tyr with Ala-X-Phe [26], [27]. Kinase assays showed that p38MAPK, JNK or ERK mutants were no longer phosphorylated by their associated MKKs (Fig. 3D, 3E and 3F).

### MKK3 Binds to p38MAPK

**Figure 4:**
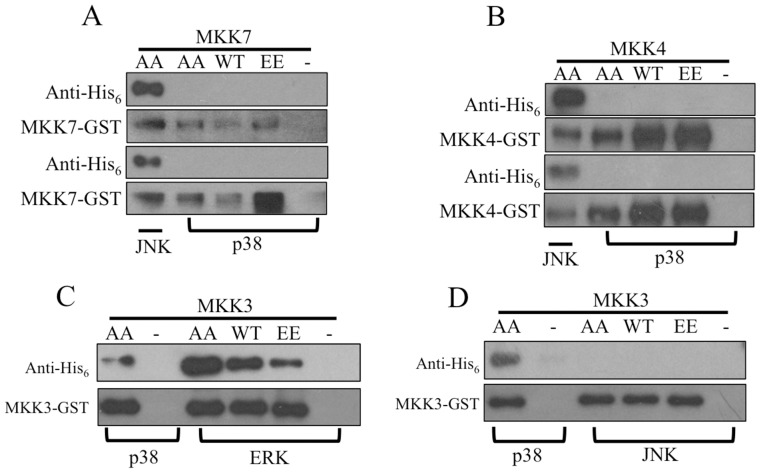
Binding dynamics might form the basis of MKK specificity. To further check the specificity of the interactions between MKKs and MAPKs, binding assays were conducted. MKKs and MAKPs were detected using antibodies against GST and His_6_ tag, respectively. Negative control, which included MAPK alone with GST beads, and positive control, which included MKK protein interacting with a known MAPK partner, were conducted simultaneously. (A) MKK7 did not bind p38MAPK. (B). MKK4 did not bind p38MAPK. (C) MKK3 bound ERK. (D). MKK3 did not bind JNK.

To better understand the specificity of the interaction between MKKs and p38MAPK, binding assays were performed. The results showed that MKK4 and MKK7 did not bind p38MAPK (Fig. 4A and 4B). MKK3 not only interacted with p38MAPK, but also ERK. The affinities between MKK3 and p38MAPK or ERK appeared to be similar (Fig. 4C). Alternatively, MKK3 did not bind to JNK (Fig. 4D).

### MKK3 and pMKK3 Mainly Localized to the Antennules of Cyprids

**Figure 5:**
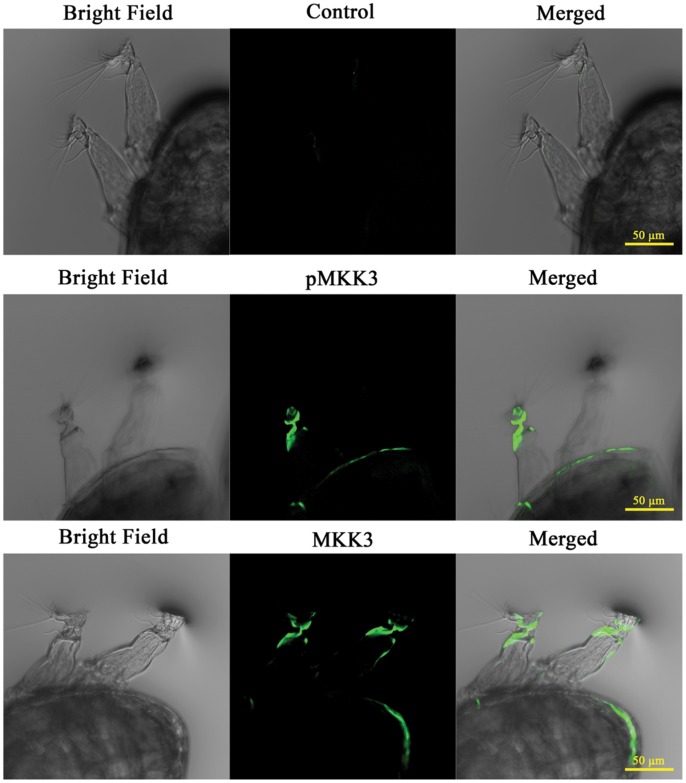
MKK3 and pMKK3 mainly localized to the antennules of barnacle cyprids. Cyprids were stained with MKK3 and pMKK3 antibodies. Larvae stained with secondary antibody alone were used as control. Images were obtained using a laser scanning confocal microscope with a 20X objective. Both MKK3 and pMKK3 were mainly concentrated at the third segment and the attachment organ of antennules.

It has been shown that p38MAPK and pp38MAPK localize to the third (containing the attachment organ) and fourth segments of the antennules in *A. amphitrite* cyprids [18]. To examine the localization of MKK3 and pMKK3 in cyprids, immunofluorescence imaging was performed using specific antibodies against MKK3 and phospho-MKK3 (pMKK3). The results demonstrated that both MKK3 and pMKK3 were abundantly present in the third and fourth segments of antennules (Fig. 5). For further observation, a series of Z-stack images was taken at the highest magnification with a 63X objective (Movie S1). The stained area is 'fiber-shaped'. It originates from the distal part of the second segment of antennules, passes through the third segment and bifurcates into two branches when entering the attachment organ, with each branch localizing close to the cuticular wall of the attachment organ and finally terminating at the margin of attachment disks. The width of the stained 'fiber' is about 5 µm at the widest part and 1 µm at the narrowest part (Movie S1).

### Phosphorylation Patterns of MKK3 and p38MAPK are Identical During the Barnacle Life Cycle

**Figure 6:**
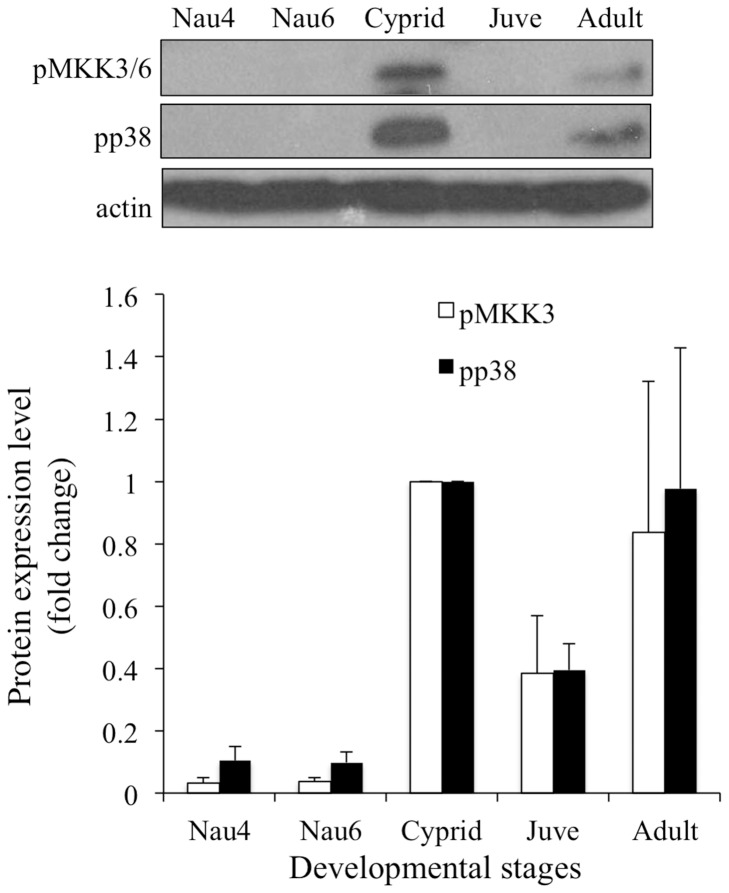
pMKK3 and pp38MAPK patterns were similar during development of *Amphibalanus amphitrite*. Equal amounts of total protein extracts (100 µg) from each developmental stage were blotted with antibodies against pMKK3 and pp38MAPK. A representative image is shown. Levels of pp38MAPK and pMKK3 in cyprid stage were normalized to a unit of 1 for relative comparison with other stages. Multiple comparison analysis showed that levels of MKK3 and p38MAPK phosphorylation were significantly elevated at cyprid stage, compared to nauplii IV, VI and juvenile stages. The levels of pMKK3 and pp38MAPK in adults did not reveal difference from those of other stages. Nau 4: stage IV Nauplii; Nau 6: stage VI Nauplii; Juve: juveniles collected 1 day after metamorphosis.

Phosphorylation levels of p38MAPK and MKK3 were investigated throughout the development of *A. amphitrite*. Levels of pMKK3 and pp38MAPK were significantly elevated in cyprids, compared to Nauplii IV, VI and juvenile stages (Fig. 6). No significant differences were observed between adults and other stages.

### Treatment with a p38MAPK Inhibitor Led to Increased Phosphorylation of MKK3 and p38MAPK

**Figure 7:**
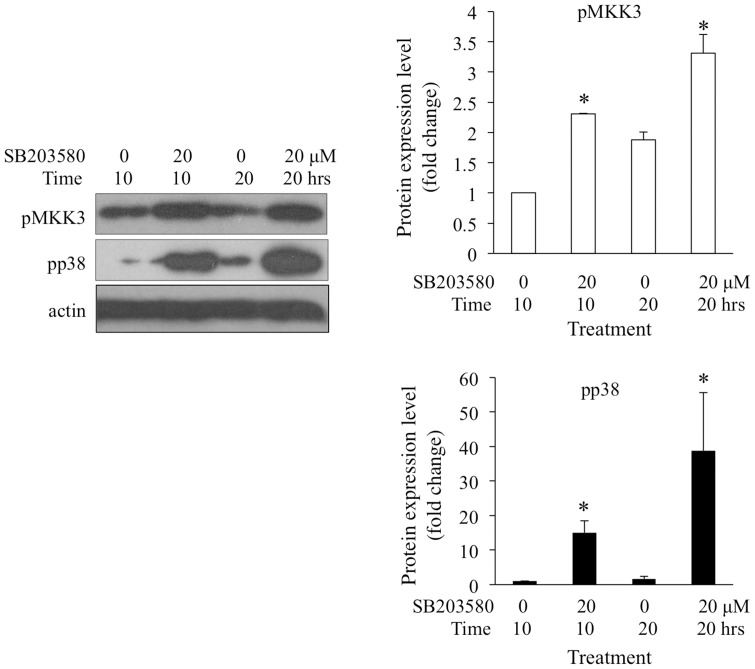
Treatment with p38MAPK inhibitor increased MKK3 and p38MAPK phosphorylation. Cyprids treated with 20 µM SB203580 for 10 and 20 hours were collected. Levels of pp38MAPK and pMKK3 were detected by Western blot. Untreated cyprids were blotted as control. The phosphorylation levels of p38MAPK and MKK3 in the 10-hour untreated group were normalized to a unit of 1. Student’s *t*-test was conducted to determine the significant levels of the differences between treated and untreated cyprids. The asterisks indicate significant differences from the untreated group within the same time course.

SB203580 is a specific p38MAPK inhibitor. Previously, the disruption of p38MAPK signaling by SB203580 was found to inhibit larval settlement of *A. amphitrite* [18]. In this study, newly transformed cyprids (within 4 hours after transformation) were treated with 20 µM of SB203580 for 10 and 20 hours. Western blotting revealed that SB203580 treatment led to increased phosphorylation of MKK3 and p38MAPK (Fig. 7).

### Crude Extracts of Adult Barnacles Activated MKK3 and p38MAPK

**Figure 8:**
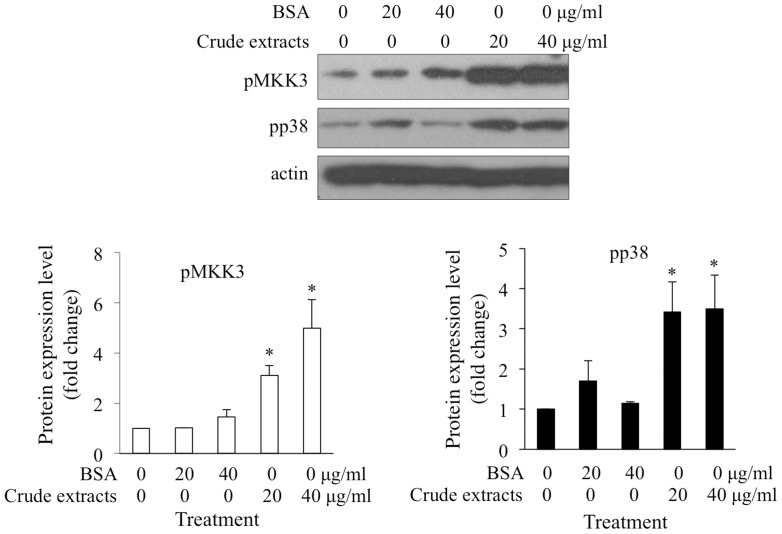
Treatment with adult barnacle crude extract significantly activated MKK3 and p38MAPK. Freshly transformed cyprids were treated with 20 or 40 µg/ml adult barnacle extract for 10 hours. The same concentration of BSA was used as protein control and cyprids without treatment were used as blank control. The relative levels of pp38MAPK and pMKK3 were determined by Western blot. Phosphorylation levels of MKK3 and p38MAPK in blank control were assigned unit of 1 and used for normalization. Multiple comparison analysis showed that both 20 and 40 µg/ml of adult barnacle extract significantly induced the activation of p38MAPK and MKK3, but the same concentration of BSA did not have any effects. The asterisks indicate significant differences from the blank control.

Treatment with adult barnacle crude extracts can induce activation of p38MAPK and larval settlement [18]. In this study, 20 and 40 µg/ml adult extracts significantly activated MKK3 and p38MAPK in cyprids, compared with untreated larvae. As a control, the same concentrations of BSA did not evaluate the activation level of MKK3 or p38MAPK (Fig. 8). These results demonstrated that MKK3-p38MAPK pathway might mediate larval settlement in response to adult extracts.

## Discussion

In mammalian systems, MAPKs are activated upon dual phosphorylation of their Thr-X-Tyr motif. In previous studies, *in vitro* kinase assays were performed using γ-P^32^-ATP and detected by autoradiography to track phosphorylation of proteins [20]–[23]. In the present study, we employed antibodies specific to the conserved and dually phosphorylated Thr-X-Tyr motif of MAPKs to assess the activities of MKKs on MAPKs. These antibodies are specific to the dually phosphorylated state of the Thr-X-Tyr motif since they do not recognize the unphosphorylated or phosphorylation-deficient Ala-X-Phe motif. Since autoradiography does not provide information at the sequence level, we employed these highly specific antibodies to follow the activities of barnacle MKKs.

MKKs are active when phosphorylated on their Ser-X-X-X-Thr motif. We obtained constitutively activated barnacle MKKs by replacing the Ser and Thr with Glu [20]–[23], which resembles the negatively charged state of phosphorylated Ser/Thr [26]. These mutants did not significantly enhance active ERK levels, compared to inactivated MKKs. It appeared that ERK was activated automatically and was insensitive to the presence of MKKs, as previously noted [24], [25].

MKK3, MKK6 [26], [28] and MKK4 [28]–[32] have been identified as p38MAPK activators in mammalian cells. In this study, kinase assays showed that MKK3, but not MKK4 or MKK7, specifically phosphorylated p38MAPK. Immunostaining showed that MKK3 and pMKK3 localized to the third and forth segments of barnacle antennules, which is consistent with the localizations of p38MAPK and pp38MAPK in *A. amphitrite* [18]. Moreover, the patterns of MKK3 and p38MAPK phosphorylation were similar during *A. amphitrite* development and in cyprids treated with adult extracts or with the p38MAPK inhibitor SB203580. This co-occurrence of pMKK3 and pp38MAPK during barnacle development, together with our kinase assay data, suggested that MKK3 and p38MAPK were interacting *in vivo*. Indeed, we found that MKK3 binds to p38MAPK with high affinity. Taken together, we conclude that MKK3 is a specific activator of p38MAPK in the barnacle *A. amphitrite*.

Substrate specificity of individual MKKs contributes to regulation of signal transduction during various biological processes [9], [11], [26], [33]. In this study, the interaction between MKK3 and p38MAPK also showed high specificity, as only MKK3 phosphorylated p38MAPK and MKK3 did not phosphorylated JNK or ERK. According to the literature, two factors may contribute to the specificity of the MKK3-p38MAPK interaction [33], [34]. One is the selective formation of functional complexes mediated by the docking sites on the N-terminus of the MKKs [33]. Another factor is the structure of the MAPK activation loop, which contains the Thr-X-Tyr dual phosphorylation motif. Results in present study indicated that MKK4 and MKK7 could not bind or phosphorylate p38MAPK and MKK3 was unable to phosphorylate JNK. Considering that the docking sites in the N-termini of MKKs are divergent in *A. amphitrite,* it is conceivable that the specificity of these kinases occurs at the level of complex formation. However, MKK3 was able to bind, but not phosphorylate, ERK. This result might be related to the structure of activation loop of ERK [10], [35]–[38], which is remarkably different from that of p38MAPK, as showed in Fig. S1.

It was previously shown that p38MAPK is important for larval settlement of *A. amphitrite* [18]. Since mammalian p38MAPK is activated by MKK3, MKK6, and sometimes MKK4 and TAB1 (transforming growth factor-β-activated protein kinase 1-binding protein) [39], or autophosphorylation [40], we checked whether MKK3 signaling was involved in larval settlement via p38MAPK activation in *A. amphitrite*. We found that pp38MAPK and pMKK3 levels were elevated at cyprid stage compared to nauplii IV, VI and juvenile stages. This indicates that p38MAPK activity may be mediated through MKK3 signaling during larval settlement of *A. amphitrite.*

During the larval settlement, antennules explore surfaces, produce a tacky secretion for temporary adhesion, hold the main body of larvae and finally attach on the surface tightly by secreting cement proteins. Antennules consist of four segments and the third segment is modified as an attachment organ with a disk by which the cyprid attaches to a surface. Internally, the attachment organ has the axially situated cement duct, two muscles, an array of glands and sense organs [41]. The sense organs can be further divided into the axial sense organ, the postaxial sense organ and the radial sense organs (two with internal tube and six without internal tube). Among these fine structures, only the two radial sense organs with internal tube emerge from the margin of the attachment disk and localize relatively close to the cuticular wall of the attachment organs. Moreover, they are fiber-sharped with about 1 µm width in the attachment organ [41]. All of these feathers are consistent with the immunostaining results obtained in this study for MKK3 and pMKK3. Based on these pieces of information, we suspected the structures immunostained by MKK3 and pMKK3 antibodies might be the radial sense organs. These radial sense organs are responsible for environmental exploration by working as both mechanoreceptors and chemoreceptors [41]. These results reminded that MKK3-p38MAPK might be involved in larval settlement by detecting and transducing the external signal. However, more evidence is needed to further confirm this point.

Normally, inhibition of MKK3 is an effective method to investigate the involvement of MKK3 in larval settlement. However, commercially available inhibitors of MKK3 are not definitely specific to this kinase, to the best of our knowledge. Alternatively, to confirm the phosphorylation of p38MAPK by MKK3 *in vivo*, reversely increased activation of MKK3 is expected in response to inhibition of p38MAPK through negative feedback regulation [42], [43]. SB203580 is widely implicated as a specific inhibitor of p38MAPK [17], [44]–[46]. It binds to an extended pocket in the active site of p38MAPK and blocks the formation of functional complexes with substrates [47]. In this study, inhibition of p38MAPK signaling significantly increased the levels of MKK3 and p38MAPK phosphorylation. Despite this, it was previously shown that SB203580 prevents larval settlement. Taken together, it suggested that MKK3 is involved in the p38MAPK pathway that controls larval settlement.

Crude extracts of barnacle adults have been used to induce larval settlement [48], [49]. In this study, both MKK3 and p38MAPK were highly activated in response to treatment with adult extracts. These results suggested that MKK3 might mediate the effects of adult extracts on larval settlement through activating p38MAPK.

### Conclusion

Previous studies have indicated that p38MAPK plays a crucial role in larval settlement of the barnacle *A. amphitrite*. Here, we performed kinase assays with recombinantly expressed barnacle MKKs and MAPKs. We found that MKK3 is a specific activator of p38MAPK. Phosphorylation of MKK3 and p38MAPK co-occurred temporally and spatially during barnacle development, suggesting that MKK3 phosphorylates p38MAPK *in vivo*. Moreover, we found that adult extract-induced larval settlement may be mediated through MKK3-p38MAPK signaling.

## Materials and Methods

### Ethics Statement

The barnacle *Amphibalanus amphitrite*, a common marine invertebrate species, is not under protection by any organization in Hong Kong. Therefore, no specific approval is required for barnacle operations. Adult barnacles in this study were scraped off from a wild population settling on concrete columns in Pak Sha Wan, Hong Kong (22°21′45′′N, 114°15′35′′E). Pak Sha Wan is a public dock and it does not belong to any national parks, protected areas or private lands, so no specific permission is required for field studies at this location. Field studies at Pak Sha Wan do not involve any endangered or protected species or contravene any environmental protection law.

The protocol for antibody generation using rabbits was approved by the Department of Health in the Government of Hong Kong, with the reference number "(12–35) in DH/HA&P/8/2/2 Pt.4", and also approved by the Animal Ethics Committee at the Hong Kong University of Science and Technology with the reference number 2012028. All precautions were taken to minimize animal suffering.

### Larval Collection and Culture

Larvae were released according to the general protocol [50] and then cultured in 0.22 µm-filtered sea water (FSW) at 24°C with the light:dark of 12 h:12 h. Larvae were fed daily with a diet of *Chaetoceros gracilis* Schutt at 1×10^6^ cells/ml until they transformed to cyprids. For collection of juveniles, cyprids were moved into clean Petri dishes. After 1–2 days, healthy and competent individuals would settle on the surfaces and further metamorphose to juveniles.

### RNA Extraction and Gene Cloning

Total RNA was prepared from cyprids using TRIzol Reagent (Invitrogen) according to the manufacturer's instructions. After trace DNA contamination was eliminated by the treatment of a Turbo DNA free kit (Ambion), cDNA was synthesized using M-MLV Reverse Transcriptase Tested User Friendly™ Kit (USB Corporation, Cleveland, OH) with oligo dT primer.

Full length p38MAPK in *A. amphitrite* (JQ277477 in Genbank) was isolated by He et al. [18]. In the present study, reciprocal blast was performed using *Drosophila* and human MKK and MAPK sequences (Table S1) against the transcriptome database of *A. amphitrite* [19]. In this way, partial sequences of barnacle MKK3, MKK4, MKK7, JNK and ERK were obtained (Table S2). Primers were designed based on these sequences to obtain the full length sequence for each gene, which were subsequently used to clone MKK3, MKK4, MKK7, p38MAPK, JNK and ERK. All the sequences were deposited to Genbank database with the accession numbers listed in Table S2. All the primers used in this study were listed in Table S3.

### Sequence Analysis

The MAPKs and MKKs were aligned separately, using the online Clustal W2 tool (http://www.ebi.ac.uk/Tools/msa/clustalw2), and the subdomains were divided according to He et al. [18] and Raingeaud et al. [26], respectively.

The phylogenetic tree was constructed based on the protein sequences of MKK3 from different species using the Maximum Likelihood method and the MEGA 5.10 program [51]. The p38MAPK from *A. amphitrite* was included as an outgroup and the support for each node was tested with standard bootstrap analysis through 1,000 replications.

### Plasmid Construction and Expression of Recombinant Proteins

To create constitutively activated forms, Ser-253 and Thr-257 of MKK4 [MKK4(EE)], Ser-208 and Thr-212 of MKK3 [MKK3(EE)] as well as Ser-316 and Thr-320 of MKK7 [MKK7(EE)] were mutated to Glu using overlap PCR. The same sites were mutated to Ala to construct constitutively inactivated forms of these proteins [20]–[23]. These mutated forms and wild type forms were inserted into the pGEX-4T vector (GE Healthcare Bio-Sciences AB, Björkgatan, Sweden) to facilitate GST-tagged protein expression.

The phosphorylation-defective mutations of JNK, ERK and p38MAPK were prepared by replacing Thr-197 and Tyr-199 of JNK, Thr-190 and Tyr-192 of ERK and Thr-177 and Tyr-179 of p38MAPK with Ala and Phe, respectively [26], [27]. These mutated sequences, together with wild type forms, were subcloned into the pET-28a vector (Novagen) for His_6_-tagged protein expression.

After these recombinant plasmids were confirmed by sequencing, they were transformed into *E. coli* BL21 (DE3) strains for expression. Recombinant proteins with GST or His_6_ tags were purified using GST-Sepharose (Sigma, USA) or Ni^2+^-nitrilotriacetic acid beads (Qiagen, USA), respectively. Special centrifugal filters of regenerated cellulose (Amicon Ultra, Millipore, Iceland) were employed to concentrate the target proteins, and then the purity of these proteins was checked by SDS-PAGE. The protein concentrations were determined using the RC-DC method (Bio Rad, USA).

### Antibody Generation

To generate barnacle MKK3 antibody, a fragment from amino acid (aa) 1 to aa 102 was subcloned into both the pET-28a and pGEX-4T vectors to produce recombinant proteins fused with His_6_ tag and GST tag, respectively. Detailed protocols for antigen injection and antibody purification have been previously described by Fong et al. [52]. Similarly, to get barnacle MKK3 antibody, His_6_-tagged recombinant proteins were injected intracutaneously with Freund's adjuvant (Sigma, USA) on the back of New Zealand white rabbits up to 4 times, and the whole blood was collected 1 week after the final injection. GST-tagged proteins were immobilized on 0.22 µm PVDF membrane (Millipore, USA) and subsequently used as bait to isolate the MKK3 antibody from the serum.

Phospho-p38MAPK (pp38MAPK, Thr180/Tyr182), phospho-ERK (pERK, Thr202/Tyr204) antibodies as well as anti-rabbit and anti-mouse HRP-labeled secondary antibodies were purchased from Cell Signaling Technology (USA). Phospho-MKK3 antibody (pMKK3, Ser189) and phospho-JNK (pJNK, Thr183/Tyr185) were purchased from Santa Cruz Biotechnology, Inc (USA). β-actin antibody and anti-rabbit Alexafluor488-labeled antibodies were purchased from Millipore (USA) and Invitrogen (USA), respectively.

### Kinase Assays

Kinase assays were performed with 2 µg of GST-MKK, 4 µg of His_6_-MAPK and 20 µM ATP in a final volume of 50 µl of kinase buffer (20 mM HEPES, pH 7.6, 20 mM MgCl_2_, 2 mM DTT, 0.1 mM Na_3_VO_4_ and commercial protease inhibitors and phosphatase inhibitors (Roche, Germany)). After being incubated at 30°C for 30 min, reactions were terminated by addition of Laemmli sample buffer. The phosphorylation results were determined using Western blot against pp38MAPK, pJNK or pERK. Anti-His_6_ antibody was used to evaluate loading per lane.

### Binding Assays

10 µg of GST-MKK was incubated with 20 µl of GST beads in 500 µl of binding buffer (1×PBS, pH7.4, 1 mM EDTA, 1 mM DDT, 100 mM NaCl, 0.2% Triton-X100, commercial protease inhibitors and phosphatase inhibitors) at 4°C for 30 min. The beads were washed with binding buffer for 5×5 min, then further incubated with 10 µg of a His_6_-MAPK at 4°C overnight. After being washed for 5×5 min, the beads were boiled in 1×Laemmli sample buffer for 20 min and subjected to 12% PAGE-gel for Western blot. The bound proteins were examined using anti-His_6_ antibody.

### Immunofluorescence Imaging

Cyprids were relaxed in 0.37 M MgCl_2_ for 15 min and then fixed in 4% paraformaldehyde (PFA) in PBS solution at 4°C until use. For immunostaining, cyprids were treated with 0.5% Triton X-100 in PBS for 30 minutes at room temperature, blocked with 5% bovine serum albumin (BSA) in PBS, and incubated with primary antibodies against MKK3 or pMKK3 using 1∶200 dilution overnight at 4°C. After being washed with PBS for 3×15 min, the larvae were further incubated with the Alexafluor488-labeled anti-rabbit IgG antibody using 1∶1000 dilution at 4°C for 12 hours. Cyprids stained the secondary antibody alone were used as the control. Images were observed under a laser scanning confocal microscope (Zeiss, LSM 710, ZEN 2009 software, USA).

### Protein Extraction and Western Blot

To examine the levels of pMKK3 and expression of MKK3 during development, Nauplii IV and VI, cyprids and juveniles were collected during larval culture. Healthy adults were selected from the fields directly.

To test the negative feedback effects of p38MAPK inhibition on the activation of MKK3, newly transformed cyprids (within 4 hours after transformation) were treated with 20 µM SB203580 for 10 and 20 hours, and then collected for Western blot.

To test whether the effects of adult extracts on p38MAPK activation are mediated by MKK3, crude extracts of adults were prepared by homogenizing barnacle adults in FSW. Cyprids treated with 20 or 40 µg/ml crude extracts for 10 hours were collected. Same concentrations of BSA were used as protein controls and untreated larvae were collected as blank control at the same time. After collection, all of these samples were rapidly frozen in liquid nitrogen until use.

Barnacle larvae or adults were lysed in lysis buffer (8 M Urea, 40 mM DTT, commercial protease inhibitors and phosphatase inhibitors) by sonication for 3×20 sec (Branson Digital Sonicator 250, Danbury, CT). Debris was discarded after centrifugation at 15 k×g for 20 min. The RC-DC method (Bio Rad, USA) was used to measure the protein concentration of each lysate. Crude lysates (120 µg) were separated on 12% SDS-PAGE gel and then transferred onto PVDF membrane (0.22 µm pore size, Millipore, USA). Antibodies against actin, pp38MAPK and pMKK3 were used as primary antibodies. Actin was used as loading control.

All the Western blot experiments were repeated at least three times. The intensity of the blotting signal were analyzed with the Quantity One software (Bio-Rad, USA), and represented as fold changes. Statistical analysis was conducted with SPSS 11.5 software.

## Supporting Information

Figure S1 **The alignments of MKKs and MAPKs in** ***Amphibalanus amphitrite*** **.** The full-length coding regions of MKKs and MAPKs were aligned using the ClustalW2 tool. The dual phosphorylation sites of these genes were labeled with asterisks and the subdomains I to XI were indicated with Roman numbers. (A). The alignment of three MKK genes. The N-termini are different among the three MKK genes, which is highlighted in the blue box. (B) Alignment of JNK, ERK and p38MAPK. The activation loops of the three MAPK genes are labeled with a red box.
(TIF)

Table S1 **The fly genes from** ***Drosophila*** **and human used to identify MAKPs and MKKs in the** ***Amphibalanus amphitrite*** **transcriptome database.**
(TIF)

Table S2 **The contig numbers of MAPK and MKK genes in the** ***Amphibalanus amphitrite*** **transcriptome database and Genbank access numbers for the complete coding regions of these genes.** By blasting against the *A. amphitrite* transcriptome database using human or *Drosophila* genes, several MKK and MAPK homologs were identified in *A. amphitrite*. After RACE reactions, the complete coding regions of these genes were identified and deposited into Genbank.
(TIF)

Table S3 **Primers used in this study.**
(TIF)

Movie S1 **Z-stack results of the inmmunostaining area for pMKK3.** Cyprids stained for pMKK3 in Figure 5 were further examined at the highest magnification with a 63X objective. The Z-stack images were taken at an interval of 0.2 µm for a total height of 34 µm.
(RMVB)
