# Supplementary figures and images for: MKK3 Was Involved in Larval Settlement of the Barnacle Amphibalanus amphitrite through Activating the Kinase Activity of p38MAPK
Source: PLoS One. 2013 Jul 29;8(7):e69510. doi: 10.1371/journal.pone.0069510 (PMC3726695; doi:10.1371/journal.pone.0069510)

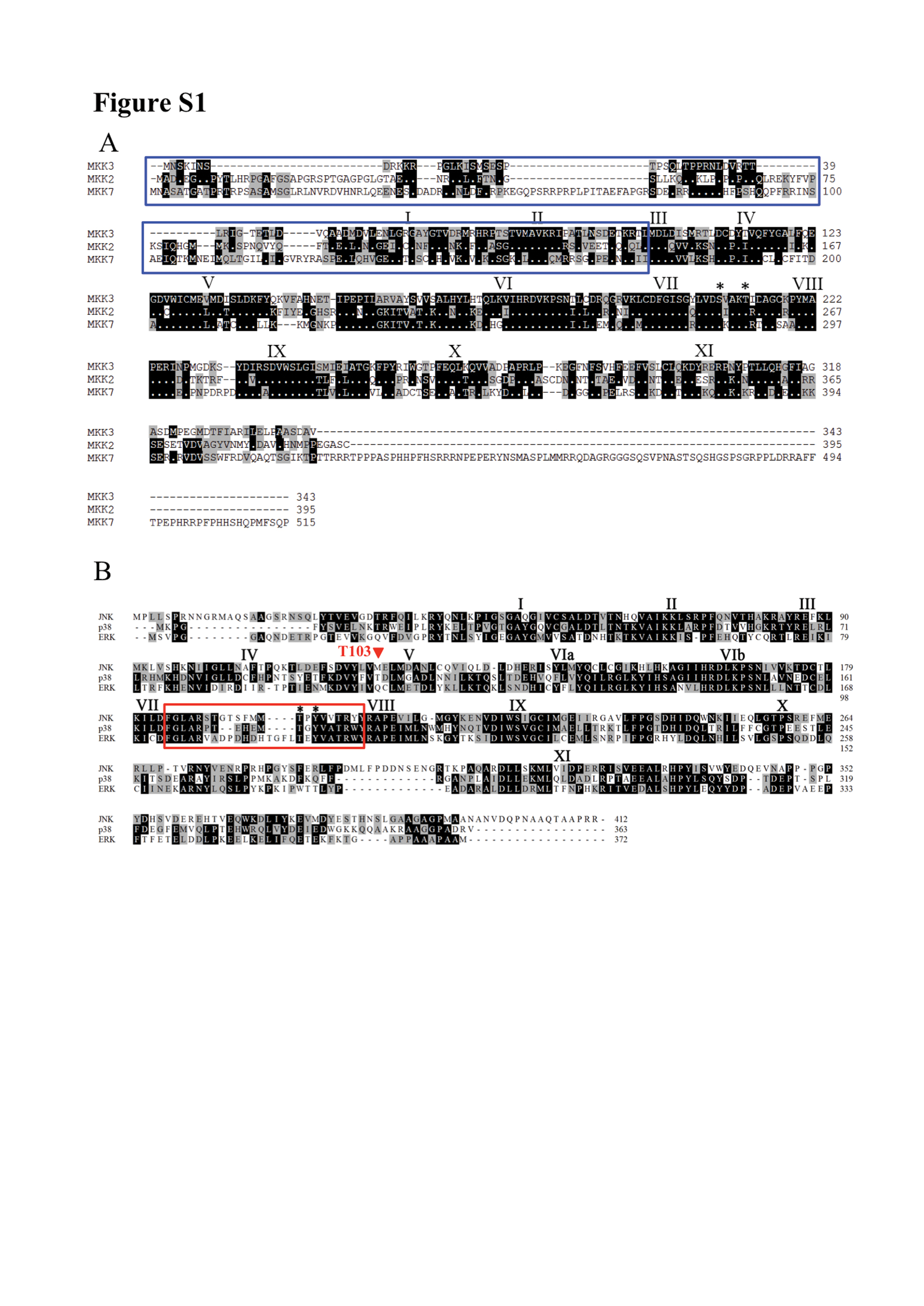

Supplement: Figure S1 — The alignments of MKKs and MAPKs in Amphibalanus amphitrite . The full-length coding regions of MKKs and MAPKs were aligned using the ClustalW2 tool. The dual phosphorylation sites of these genes were labeled with asterisks and the subdomains I to XI were indicated with Roman numbers. (A). The alignment of three MKK genes. The N-termini are different among the three MKK genes, which is highlighted in the blue box. (B) Alignment of JNK, ERK and p38MAPK. The activation loops of the three MAPK genes are labeled with a red box. (TIF) [file pone.0069510.s001.tif]

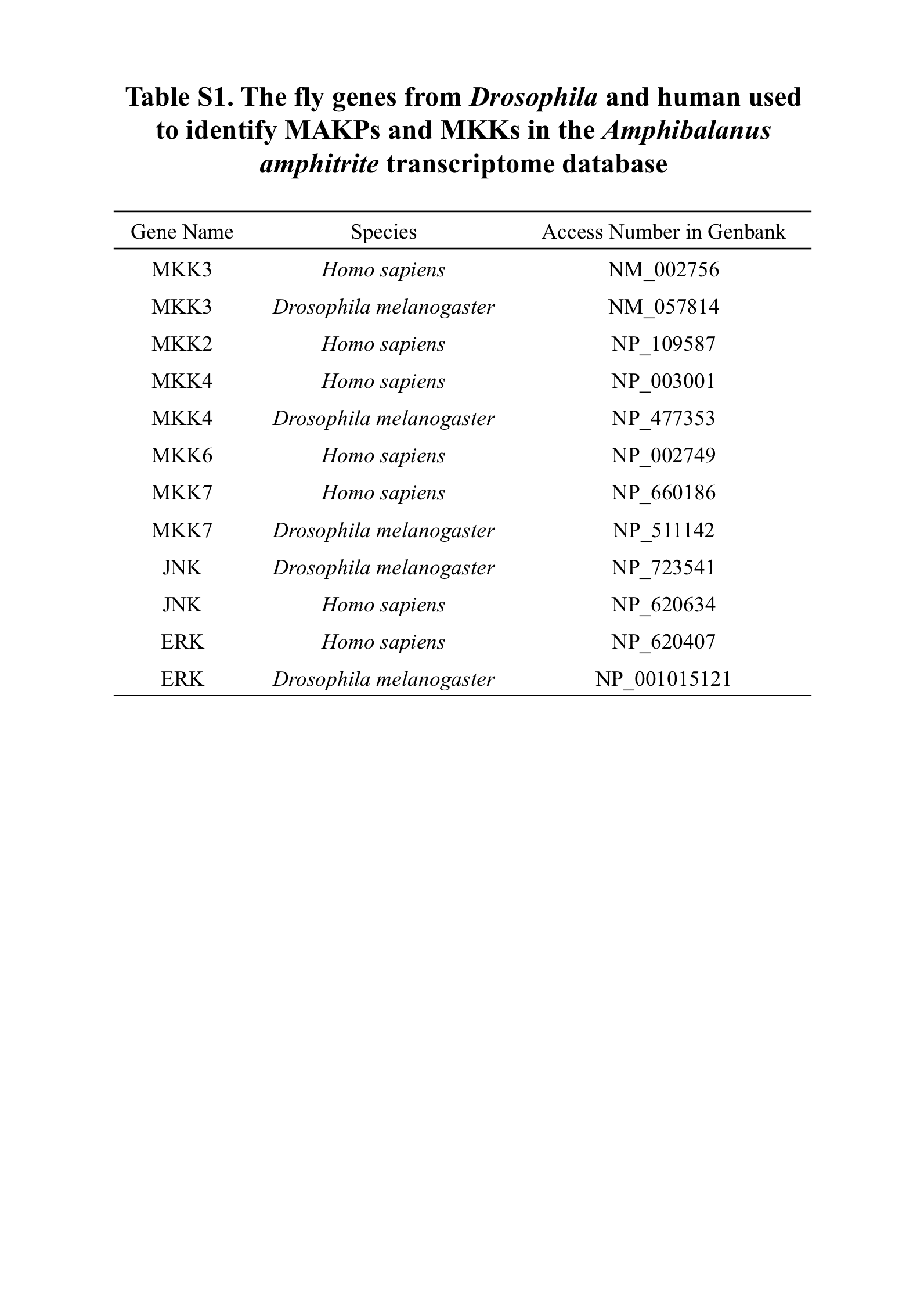

Supplement: Table S1 — The fly genes from Drosophila and human used to identify MAKPs and MKKs in the Amphibalanus amphitrite transcriptome database. (TIF) [file pone.0069510.s002.tif]

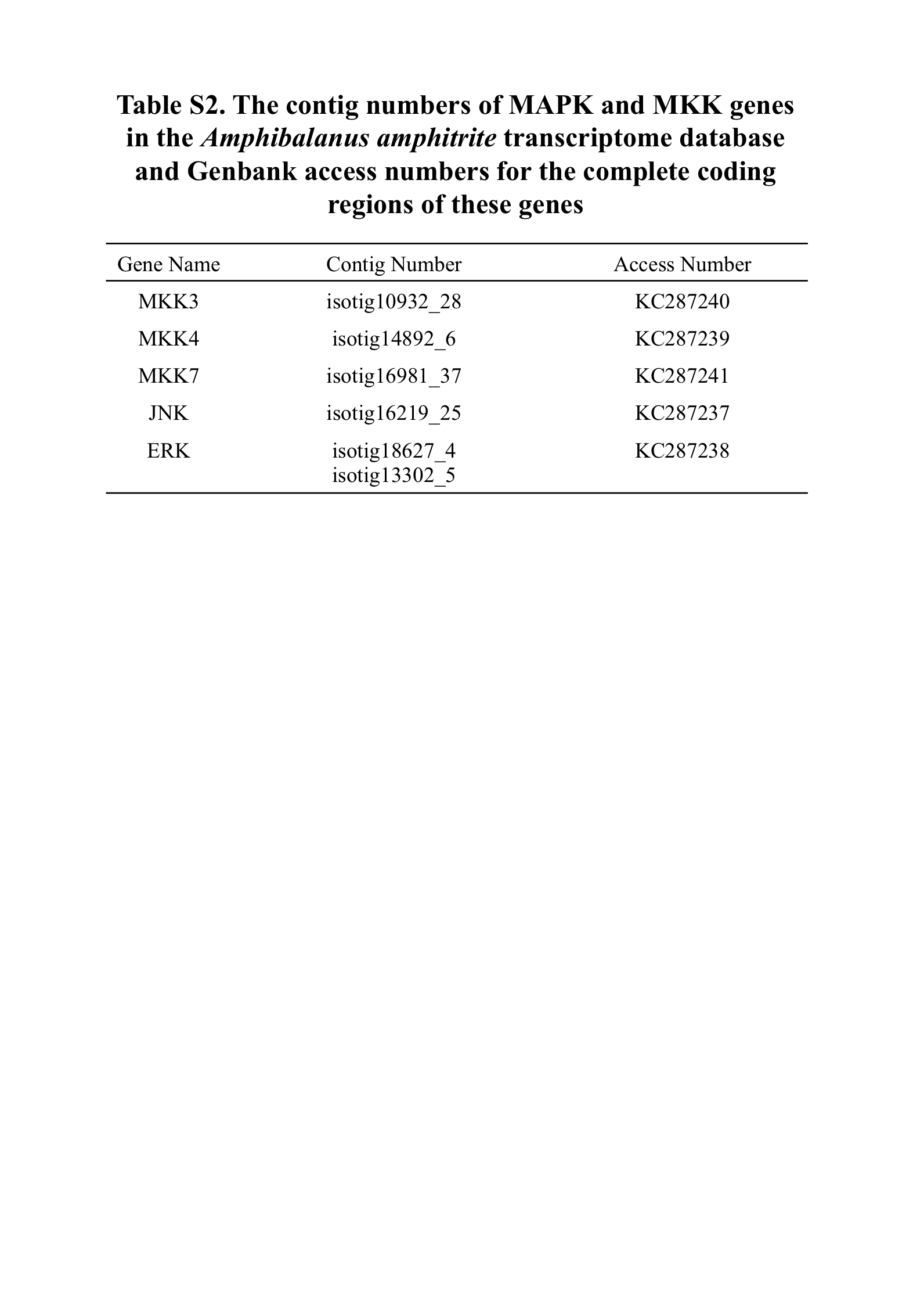

Supplement: Table S2 — The contig numbers of MAPK and MKK genes in the Amphibalanus amphitrite transcriptome database and Genbank access numbers for the complete coding regions of these genes. By blasting against the A. amphitrite transcriptome database using human or Drosophila genes, several MKK and MAPK homologs were identified in A. amphitrite. After RACE reactions, the complete coding regions of these genes were identified and deposited into Genbank. (TIF) [file pone.0069510.s003.tif]

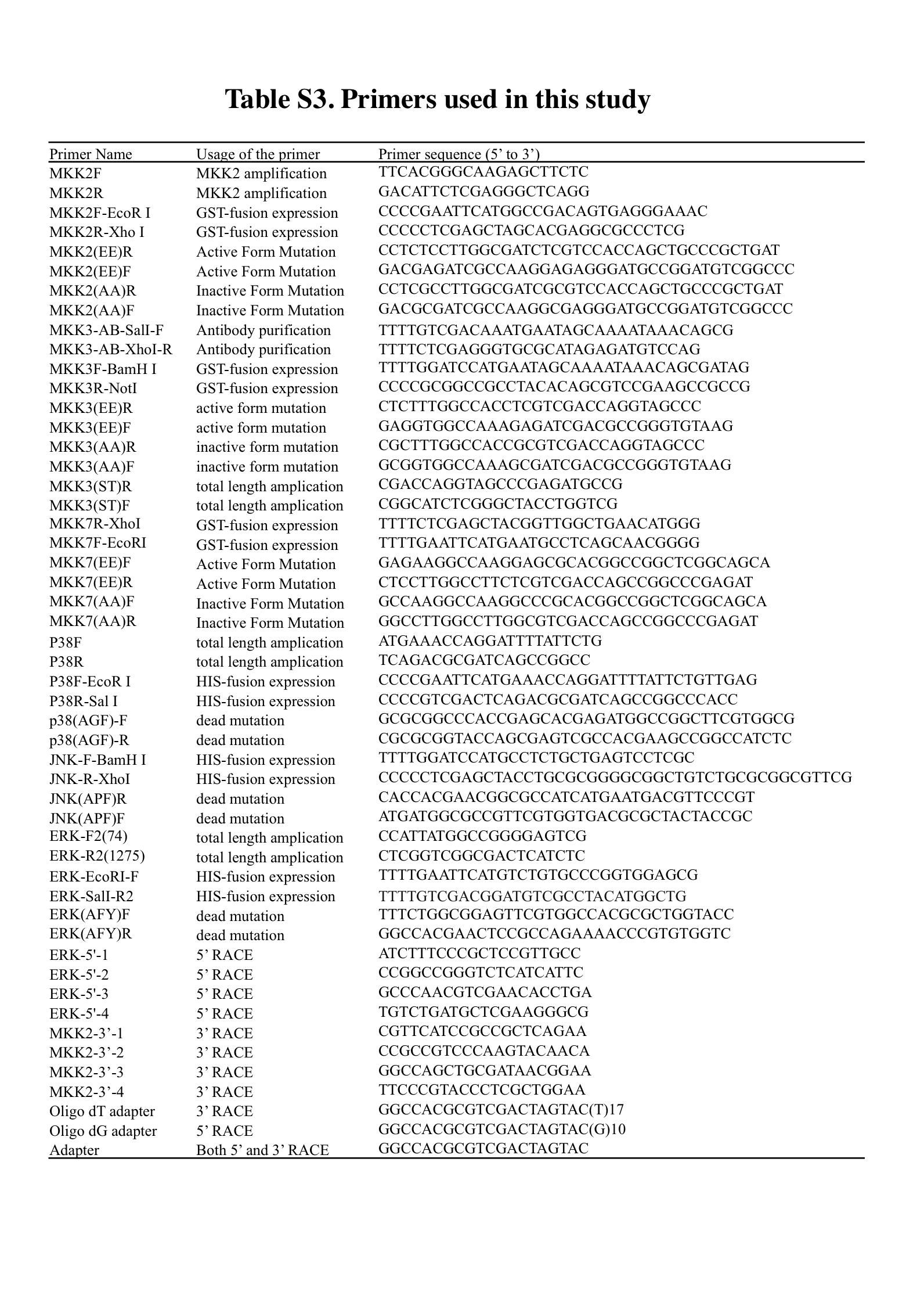

Supplement: Table S3 — Primers used in this study. (TIF) [file pone.0069510.s004.tif]
